# Dairy farmer’s perception on feeding, conservation, and constraints of brewery by-products utilization in selected districts of Ethiopia

**DOI:** 10.1016/j.heliyon.2022.e12769

**Published:** 2022-12-30

**Authors:** Geberemariyam Terefe, Getu Kitaw, Mesfin Dejene, Dereje Fekadu, Aemiro Kihalew, Bethlehem Mekonnen, Mulugeta Walelgne

**Affiliations:** Ethiopian Institute of Agricultural Research, P.O. Box 2003, Addis Ababa, Ethiopia

**Keywords:** Brewery by-products, Conservation, Constraints, Feeding, Spoilage

## Abstract

A survey was conducted in Ada’a, Sululta, and Debre Birhan districts. The districts are located in the vicinity of brewery factories. A semi-structured questionnaire was used to collect data from purposively selected dairy farmers (160). Data were analyzed with a statistical package for social sciences (Version 21). The majority of farmers (69.4%) used wet brewery-spent grain (WBSG), whereas 30% of them used both WBSG and wet brewery spent yeast (WBSY). Farmers obtained WBSG and WBSY only in fresh form from the distributors. The majority of farmers (66.67%) blended WBSG and WBSY with concentrate and roughage feed before feeding it to their animals, while 14.47% fed the by-products alone to their animals. Several farmers (60.1%) responded that the key reason for providing WBSG and WBSY to their livestock was higher production (increased milk and growth rates). The majority (82.78%) of farmers used common salt to extend the shelf life of WBSG and WBSY. Out of 128 (80%) farmers who reported spoilage in WBSG, 49 (38.28%) farmers observed sever mold development, while the remaining 12 (9.38%) and 28 (21.88%) saw change in colour and unpleasant odor. According to 68 (53.13%) of the farmers who experienced in WBSG spoiling, the amount of spoilt was less than 9% and 10–20% of the total purchased. The majority of farmers (87.8%) reported that storage time and storage conditions (temperature, moisture, and humidity) were the primary reasons of WBSG spoilage, whereas 12.2% of the farmers reported that inadequate sanitation of feeding troughs, transportation, and storage facilities were the primary causes of spoilage. The key restrictions of brewery by-product utilization were found as scarcity and high purchasing costs. Farmers (44.38% and 41.86%) believed that feeding WBSG and WBSY to dairy cattle have negative health effect, respectively. In conclusion, insufficient and irregular supply, rising cost of material and transport, spoilage, and health-related hazards are the main constraints of WBSG and WBSY usage. It is suggested that there is a dire need for consistent supply, staying away from the brokers, and preserving the brewery by-products through sun drying, and ensiling. Additionally, more research is required to determine the negative health impact of feeding brewer by-products for dairy cattle.

## 1 Introduction

The use of alternative non-conventional feed resources such as feeds from the brewery and sugarcane factories could provide practical and sustainable solutions to the prevailing crises in livestock feed scarcity. The potential availability, in 2016/17 alone, the total annual brewers’ spent grain (BSG) and brewers’ spent yeast (BSY) production from twelve running beer factories in Ethiopia was estimated at 26,723 tons dry matter (DM) and 360,758 hector liters, respectively [1].

BSY is the second largest but the least used by-product feed from the brewing industry [2]. It is an effective animal feed because it contains high levels of protein, minerals, and vitamins and boosts the fat and total solids content of cow milk [3,4].

BSG is contain high level of protein 27–33% [5], and 40–56% on DM basis [6], and an excellent source of B-complex vitamins, nucleic acids, vitamins, and minerals [6]. Low dry matter content, which impedes transport and storage, is the primary limiting issue for the efficient use of BSY and BSG, but it can be remedied by drying and ensiling [7]. The dairy farmers do not efficiently use the BSY and BSG, even when they are close to the factories [1]. The main problem to adopt agro-industrial by-products is microbial infestation [8].

Brewer by-products have promising nutritional benefits for dairy cattle, but the hindrances and prospects of using the BSY and BSG as dairy cattle feed in Ethiopia have not been fully explored. This survey study was designed to close the gap by assessing the BSY and BSG mode of feeding, conservation, hygienic, and storage procedures, spoilage, and health-related issues affecting to the dairy cattle.

## 2 Materials and methods

### 2.1 Description of the study areas

**Figure (1):**
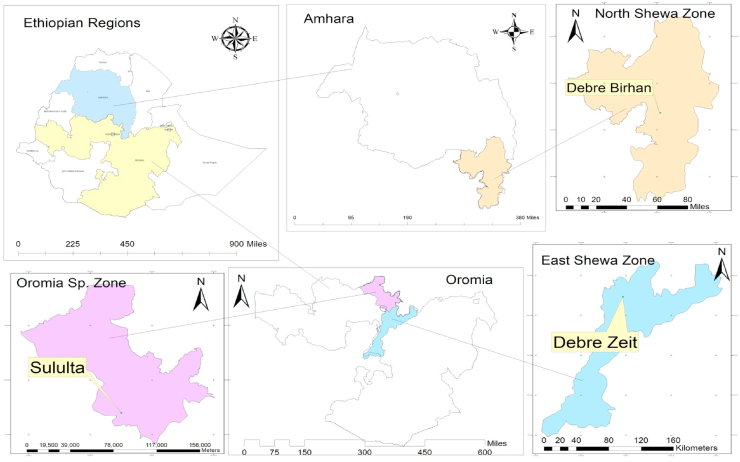
Map of study areas.

The survey was conducted in the two districts of Oromia (Debr Zeit town/Ada’a and Sululta) and one district (Debre Birhan) in Amhara regional states of Ethiopia (Fig. 1). Debr Zeit town is found Ada’a district of Oromia regional state and located in 08° 44’ N latitude and 38° 58’ E longitude with average altitude of 2053 m above sea level (m.a.s.l). The average temperature is 19 °C and the area receives 851 mm precipitations annually. Sululta district is found in a special zone of Oromia and located in 09° 11’ N latitude and 38° 45’ E longitude with average altitude of 2588 m. a.s.l. The average annual rainfall of the area is 1232 mm and with average temperature of 16^o^c. Debre Birhan district is found in a North shoa zone of Amhara regional sate and located in 09° 12’ N latitude and 38° 45’ E longitude with average altitude of 2840 m. a.s.l. The average temperature is 13 °C and the area receives 1232 mm precipitations annually. This district has two brewery factory including Habesha and Dash beer factories.

### 2.2 Sampling techniques and sample size

The respondents were chosen using a two-stage random and purposive selection method. With regard to the districts, commercial and smallholder dairy farm owners, a discussion has been held in the first stage with the district agricultural extension officers. The districts were designated based on their proximity to the brewery and the presences of dairy farms. The dairy producers were chosen from the entire population after the study areas had been determined. Simple random sampling was used to choose the sampled respondents. The sampled respondents in DebreZeit town, Sululta, and Debre Birhan districts were 40, 58, and 62, respectively. All respondents (160 farmers) involved for this study were selected on the bases of having cross bred dairy cows (2–5 crossbred dairy cows for small holder dairy farmers and (>5 crossbred dairy cows for commercial dairy farm [9], long experience on feeding of brewery by-products, strong record-keeping, and above all respondents have full commitment during the entire survey work. The sample size of the dairy farmers were determined by Ref. [10] formula (1),

(1) $$n=\frac{N}{1+N\left(e^{2\;}\right)}$$

Where: n - the sample size, N - The population size, and e − The level of precision (e = 0.05). The total dairy farmers in the study districts were 268, and 5% precision was used

(2) $$n=\frac{268}{1+268\;\left(0.05^{2\;}\right)}=160$$

### 2.3 Data collection

A semi-structured questionnaire were designed to collect household information, herd composition, and mode of feeding, spoilage, conservation, challenges, and the effect of feeding by-products on dairy cattle. Prior to data collection, the questionnaires were pre-tested, and respondents correctly comprehended and answered the questions. The questionnaire was written in English and translated into the local language (Amharic) by the researcher during the interview. The researcher then conducted an on-farm survey investigation across the study regions.

### 2.4 Data analysis

The acquired data were categorized into similar groups before being examined using SPSS (Statistical Package for Social Sciences), version 20 [11]. The chi-square test is used to examine frequency distributions (proportions) in categorical data. The one-way analysis of variance (ANOVA) approach of a general linear model was used to compare the mean of herd composition, productive and reproductive performances of cattle in the research region.

Least significance difference (P < 0.05) was used to compare the means. The model used for analysis of data was: Y_ij_ = μ + D_i_ + e_ij;_ Where, Y_ij_ = measured response variable (cattle type/herd composition, productive and reproductive performances) ith in district; μ = grand mean; Di = effect of district i; e_ij_ = random error.

Brewery by-product usage constraints were investigated using indices (weighted averages), and the aggregate ranking was obtained using a formula (3).

(3) $$\frac{sum\;of\;\left(\left(3\times Number\;of\;responces\;first\;rank+2\times Number\;of\;responces\;second\;rank+1\times Number\;of\;responces\;third\;rank\right)\right)}{\left(1\times total\;responces\;first\;rank+2\times total\;responces\;second\;rank+3\times total\;responces\;third\;rank\right)}$$

## 3 Results and discussion

### 3.1 Socio-economic characteristics of respondents

The socioeconomic characteristics of respondents are presented in Table 1. Male and female respondents were included randomly but the majority of the respondents (P < 0.05) were males. As compared with Sululta district and DebreZeit town at Ada’a district the majority of the respondents (P < 0.03) in Debre Birhan district were males and with good educational status. Socio-economic parameters are essential to determine the physical work of a farmer [12]. In this study the majority respondents (P < 0.03) are males, which is consistent with the findings of [13] who found that a substantial number of dairy producers in the Gondar regions were males. Several respondents (95.6%) were between the ages of 20 and 40 years, indicating that the majority of them were still in their productive years. This survey result contradicts the previous findings of [1,14]; who found that a significant number of dairy farmers under the age of 50. The educational background of the respondents was significantly (P < 0.000) different across the study areas, only 8.1% of them were not educated, this result does not agree with the finding of [14] in Dilla district and [13] in the Gondar district, reports about half of the dairy farmers are illiterate and [15], also reported that dairy farmers (31.1%) in Raya Kobo districts were illiterate.

**Table 1:** Socioeconomic characteristics of respondents in different districts.

Parameters		Debre Birhan	Sululta	DebreZeit	Overall	X^2^	*P*-value
		N (%)	N (%)	N (%)			
Sex	Male	59 (95.2)	47(81)	37(92.5)	143(89.4)	6.85	0.03
	Female	3(4.8)	11(19)	3(7.5)	17(10.6)		
Age	20–25	8(12.9)	8(13.8)	7(17.5)	23(14.4)	12.21	0.142
	26–30	20(32.3)	18(31)	12(30)	50(31.25)		
	36–40	31(50)	27(46.6)	21(52.5)	79(49.34)		
	>40	2(3.2)	5(8.6)	0(0)	7(4.4)		
Respondent type	Husband	44(71)	24(41.4)	32(80)	100(62.5)	18.56*	0.001
	Wife	12(19.4)	21(36.2)	4(10)	37(23.1)		
	Son	6(9.7)	13(22.4)	4(10)	23(14.4)		
Educational Background	0	5(8.1)	7(12.1)	4(10)	16(10)	29.08	0.000
	1	22(35.5)	30(51.7)	7(17.5)	59(36.9)		
	2	32(51.6)	20(34.5)	18(45)	70(43.8)		
	3	3(4.8)	1(1.7)	11(27.5)	15(9.4)		

Not able to read and write = (0), able to read & write through informal sources (adult education, religious schools, etc. (1), Grade 8–12 = (2) and Above Grade 12 = (3), χ2 = Chi-square; * = significant if p < 0.05 level of significance.

### 3.2 Herd size and composition

As shown in Table 2, the average numbers of lactating cows, dry cows, heifers, bulls, and calves per household are 6.76, 3.81, 3.71, 1.38, and 3.35, with the overall mean of 3.89, respectively. In the DebreZeit town at Ada’a district, farmers have a higher (P < 0.001) average herd size (14.15) than Debre Birhan and Sululta districts. The milk yield (litter/day) and service per conception (months) were significantly (P < 0.001) affected by the district. A higher milk yield was obtained in the DebreZeit town at Ada’a district than in the Debre Birhan, and Sululta districts. Service after calving of the cow was shorter in the Sululta district than in DebreZeit town, and Debre Birhan district. In this finding, the number of services per conception in dairy heifers is smaller than in the total mixed ration-based feeding for dairy heifers [16]. The average herd size found in this study is higher than that reported by Ref. [14] in Dilla district, Gedio zone, but, it is smaller than the report of [17] in Bahirdar district, Ethiopia. In this finding, age at first service of the heifer was longer than Boran-Fresian heifers as reported by Ref. [16] but the shorter age at first service in Jersey heifers was reported by Ref. [18]. The lower number of services per conception in the heifer was observed in the current study as compared with the report of [16] who reported a large number of services per conception in Boran-Frisian heifers, but equal number of services per conception in Jersey heifers was reported by Ref. [18].

**Table 2:** Herd size and production parameters of dairy cattle (mean ± SE) in the study districts.

Kind of cattle	Debre Birhan	Sululta	Debre Zeit	Overall	*P*-value
**Lactating cows**	3.38 ± 0.32^c^	6.76 ± 1.09^b^	16.35 ± 3.86^a^	6.76 ± 1.09	0.02
**Dry cows**	1.81 ± 0.20^c^	2.28 ± 0.73^b^	9.08 ± 2.52^a^	3.81 ± 0.73	0.05
**Heifers**	1.63 ± 0.21^b^	2.50 ± 0.62^b^	10.16 ± 3.20^a^	3.71 ± 0.75	0.01
**Bulls**	0.69 ± 0.13^b^	1.25 ± 0.19^b^	3.36 ± 0.64^a^	1.38 ± 0.17	0.05
**Calves**	1.62 ± 0.31^c^	2.84 ± 0.38^b^	7.05 ± 3.00^a^	3.35 ± 0.75	0.001
**Average herd size**	1.82 ± 0.16^b^	2.57 ± 0.5^b^	14.15 ± 4.57^a^	3.89 ± 0.77	0.001
**Production parameters**					
**Milk yield (kg/day)**	12.67 ± 0.56^b^	11.82 ± 0.48^b^	15.12 ± 0.62^a^	12.98 ± 0.33	0.001
**Lactation length(months)**	8.31 ± 0.26	8.28 ± 0.20	7.98 ± 0.26	8.22 ± 0.14	0.06
**No of service per conception**	1.90 ± 0.09^b^	1.72 ± 0.08^b^	2.40 ± 0.09^a^	1.96 ± 0.07	0.000
**Age at first service (months)**	18.23 ± 0.66	21.36 ± 0.72	17.40 ± 0.81	19.16 ± 0.44	0.06
**Service after calving (months)**	4.36 ± 0.40	3.33 ± 0.40	3.67 ± 0.29	3.92 ± 0.21	0.06

Means in each row with different letters have a significance difference at (P < 0.01), n = number of household respondents.

### 3.3 Types of brewery by-products supplied by the distributors

As shown in Table 3, all respondents know brewery by-products and the majority of the respondents (69.4%) were used wet brewery spent grain (WBSG) and the remaining farmers (30%) were used both WBSG and wet brewery spent yeast (WBSY). All farmers (100%) reported the distributors provided the wet/fresh form of the brewery by-products. The large numbers of farmers (59.7%) in Debre Birhan district used both WBSY and WBSY than DebreZeit town and Sululta district. Brewer’s yeast can be fed fresh (liquid form) or dried and subsequently ground [19] to provide a main source of protein, vitamins, and minerals [20]. Feeding WBSG is important to avoid the cost of drying and it is served as a replacement of forages [21].

**Table 3:** Types brewery by-products supplied by the distributors in different areas.

		Debre Birhan	Sululta	Debre Zeit	Overall	X^2^	*P*-value
		N (%)	N (%)	N (%)	N (%)		
**Do you know brewery by- products?**	Yes	62(100)	58(100)	40(100)	160(100)	2.91	0.001
	No	0(0)	0(0)	0(0)	0(0)		
**If your answer is yes, which type?**	WBSG	24(38.7)	53(91.4)	34(85)	111(69.4)	45.47	0.001
	WBSY	1(1.6)	0(0)	0(0)	1(0.6)		
	Both	37(59.7)	5(8.6)	6(15)	48(30)		
**Which forms of WBSG are supplied?**	Fresh/wet	62(100)	58(100)	40(100)	160(100)	2.91	0.000
**Which forms of WBSY are supplied?**	Fresh/wet	62(100)	58(100)	40(100)	160(100)	2.45	0.000

Wet brewer’s spent grain (WBSG); wet brewer’s spent yeast (WBSY), N = number of respondents, χ2 = Chi-square; * = significant if p < 0.05 level of significance.

### 3.4 Form and mode of feeding brewery by-products

As shown in Table 4, the majority of the farmers (55%) reflected wet brewery spent grain (WBSG) was fed to animals combined with salt, while the remaining farmers (45%) utilized the WBSG in fresh, dry, and ensiled form. The farmers (54%) used fresh wet brewery spent yeast (WBSY), whereas the remaining farmers (46%) used salted and ensiled WBSY. According to the majority of farmers' responses (66.67%), WBSG and WBSY were combined with concentrate and roughage before being fed to the animal, whereas just a few farmers (14.47%) fed WBSG and WBSY alone to their animals. More farmers (62.5%) in the Sululta district reported salt was added in WBSG than in the DebreZeit town and Debre Birhan districts. In comparison to the other districts, Sululta district has the highest percentage of farmers (26.3%) who have ensiled WBSY. The addition of WBSG (20%) into legume fodder (vetch) for silage has no effect on fermentation quality or nutritional value [22]. The respondents (50.05%) weighted the daily intake of WBSG fed to the animals while the remaining 49.5% of the respondents did not weigh the daily amount of brewery by-products fed to the animals. The current study is consistent with the findings of [1,13] who stated that dairy farmers in Sebeta, Bedele, Gondar, and Debre Birhan districts of Ethiopia used WBSG and WBSY either fresh or soaked in salt and hot water.

**Table 4:** Form and mode of brewery by-product feeding to the animals.

By-product	Form/type	Debre Birhan	Sululta	Debre Zeit	Overall	X^2^	*P*-value
		N (%)	N (%)	N (%)	N (%)		
**WBSG**	Wet/fresh	17(28.3)	18(31.6)	14(35)	49(30.62)	6.61	0.36
	Dry	1 (1.7)	1(1.8)	0(0)	2(1.3)		
	Ensiled	9(15)	8(14)	1(2.5)	18(11.5)		
	Salted	33(55)	30(52.6)	25(62.5)	88(55)		
**WBSY**	Wet/fresh	27(54)	19(33.3)	6(100)	52(46)	13.15	0.01
	Ensiled	6(12)	15(26.3)	0(0)	21(18.6)		
	Salt	17(34)	23(40.4)	0(0)	40(35.4)		
**Mode of feeding?**	By-products alone	16(69.6)	6(26.1)	1(4.3)	23(14.47)	31.62	0.000
	By- products with concentrate & roughage.	35(33)	32(30.2)	39 (36.8)	106(66.67)		
	By products with roughage	11(36.7%)	19(63.3)	0(0)	30(18.86)		
**Weight the by-product fed to animal**	Yes	41(50.6)	24(29.6)	16(19.8)	81(50.05)	9.75	0.01
	No	21(26.6)	34(43)	24(30.4%)	79(49.95)		

Wet brewer’s spent grain (WBSG); wet brewer’s spent yeast (WBSY) N = number of respondents, χ2 = Chi-square; * = significant if p < 0.05 level of significance.

### 3.5 Prioritization of feeding brewery by-products

As indicated in Table 5, the majority of respondents (93.1%) prioritized animals in feeding the brewery by-products, whereas the remaining 6.9% of farmers did not. Many farmers (60.1%) stated that higher production (increased milk yield and growth rates.) when fed brewer by-products is the key justification for prioritizing. The current finding is consistent with the findings of [23]; and [24] who claimed that prioritizing agro-industrial by-products is a sustainable management strategy, and used for dairy cattle feed.

**Table 5:** Farmer perception on the major reason of prioritization of the animals.

Questions	Responses	Debre Birhan	Sululta	Debre Zeit	Overall	X^2^	*P*-value
		N (%)	N (%)	N (%)	N (%)		
**Do you prioritize animals in feeding the brewery by- products**?	Yes	58(93.5)	51(87.9)	40(100)	149(93.1)	5.41	0.05
	No	4(6.5)	7(12.1)	0(0)	11(6.9)		
**Reasons of prioritization**	Body conditions of the animal	15(24.6)	13(22.8)	8(20)	36(22.8)	7.93	0.02
	Increased productivity (milk)	30(49.2)	37(64.9)	28(70)	95(60.1)		
	Animals that didn’t get sick	4(6.6)	3(5.3)	1(2.5%)	8(5.1)		
	Shortage of the feed	12(19.7)	4(7)	3(7.5)	19(12)		

N = number of respondents, χ2 = Chi-square; * = significant if p < 0.05 level of significance.

### 3.6 Conservation practices of brewery by-products

As shown in Table 6, the majority of the farmers (96.25%) used different conservation techniques to extend the shelf life of the brewer's product while the remaining 3.75% of the farmers did not use any conservation techniques due to lack of awareness and training. The majority of farmers (92.86%) in the Sululta district conserved WBSG with salt more than in the other areas. The majority of farmers (82.78%) used salt to extend the shelf life of the WBSG, while the remaining farmers (5.96%) dried it in the sun. This finding is corroborated by Refs. [[25], [26], [27]] who noted that salt is crucial to prevent spoilage (fewer yeast and mold microbial populations) of WBSG and to improve *in situ* dry matter and crude protein degradability. According to farmers (100%), adding salt to WBSY is beneficial for increasing palatability and storage time. Similarly, [1]; and [13] reported that WBSG is preserved by dairy producers through sun drying and ensiling. In contrast to the current study [28], found that salt (NaCl) and NaOH had no favorable effect on WBSG preservation. For the majority of farmers (68.18%), the sources of information about conservation techniques of WBSG and WBSY were the neighboring farmers while few farmers (4.55%) were getting information from the factory/supplier. An extension service on the conservation of WBSG and WBSY was lower in the DebreZeit town at Ada’a district than Debre Birhan and Sululta districts. This result is in line with the finding of [1,13] who reported dairy producers were obtained information about WBSG and WBSY conservation from the neighboring dairy farmers and extension services.

**Table 6:** Conservation techniques of brewery by-products practiced by dairy farmers.

Questions	Response	Debre Birhan	Sululta	Debre Zeit	Overall	X^2^	*P*-value
		N (%)	N (%)	N (%)	N (%)		
**Do you any practice conservation techniques?**	Yes	56(90)	58(100)	40(100)	154(96.25)	9.85	0.007
	No	6(10)	0(0)	0(0)	6(3.75)		
**If “No” why not practiced?**	Lack of awareness & training	3(50)	0(0)	0(0)	3(50)	4.51	0.12
	Luck of financial capacity	1(16.66)	0(0)	0(0)	1(16.66)		
	Byproduct is daily supplied	2(33.33)	0(0)	0(0)	2(33.33)		
**If yes, which conservation technique for WBSG?**	Sun drying	5(8.93)	1(1.72)	2(5)	9(5.96)	11.36	0.02
	Silage	12(21.43)	5(8.62)	0	17(11.26)		
	Salting	35(62.5)	52(92.86)	38(90)	125(82.78)		
**If yes, which conservation technique for WBSY?**	Sun drying	0(0)	0(0)	0(0)	0(0)	2.92	0.03
	Silage	0(0)	0(0)	0(0)	0(0)		
	Salting	56(100)	58 (100)	40(100)	154(100)		
**Where did you get the experience?**	Extension services	8(14.28)	14(24.14)	4(10)	26(16.88)	27.51	0.000
	Research workers	5(8.93)	7(12.07)	4(10)	16(10.39)		
	Neighboring farmers	40(71.43)	35(60.35)	30(75)	05(68.18)		
	Factory/supplier	3(5.58)	2(3.44)	2(5)	7(4.55)		

Wet brewer’s spent grain (WBSG); wet brewer’s spent yeast (WBSY) N = number of respondents, χ2 = Chi-square; * = significant if p < 0.05 level of significance.

### 3.7 Spoilage of brewery by-products

As indicated in Table 7, the majority of farmers (80%) confirmed to have experienced in wet brewery spent grain (WBSG) spoilage; however some of them (20%) had not seen any spoilage signs. Out of 128 (80%) farmers who reported spoilage, 49 (38.28%) farmers saw significant mold development in WBSG, while the remaining farmers (31.26%) were investigated colour change and bad odor in WBSG. The other farmers (30.47%) reported that mold growth, colour changes, and unpleasant scents were indicators of WBSG deterioration. As reported by the farmers (53.13%) who encountered WBSG spoilage, the quantity of WBSG that got spoilt was less than 9% of the total delivery obtained whilst the rest of them indicated that they lost about 10–20% of the WBSG through spoilage. Out of the total received WBSG, 10% of WBSG spoilage was higher (P < 0.05) in the Debre Birhan and Sululta districts than DebreZeit town at Ada’a district while 11–20% of spoilage was higher (P < 0.05) in Debre Birhan district than the other studied area. In line with the present finding [29], noted that the majority of respondents (81.8%) revealed that less than 9% of WBSG had incurred spoiling. The majority of the farmers (69.38%) encountered spoilage of WBSY but some of them (20%) said that they were not recorded spoilage in WBSY. Out of 111(69.38%) farmers who stated spoilage in WBSY, 50.45% of them indicated that spoilage was to the extent of mold growth and the other farmers (19.82%) reported that there was investigated change in colour and bad smell in WBSY while the remaining 33(29.73%) farmers said both mold growth, colour change, and bad smells as signs of spoilage in WBSY. Of the 35(31.53%) farmers who encountered WBSY spoilage, the quantity of WBSY that got spoilt was less than 9% of the total delivery whilst the rest of them (73.87%) indicated that they lost about 10–20% of the WBSG through spoilage, this result coincides with the findings of, [29]; who noted that all of the farmers revealed that WBSY had spoiled. The risk of spoiling exists when brewer spent grain is delivered by open truck along with other products [30]. The problems of using WBSG as fresh feed is the presence of higher moisture content and easy deterioration between 3 and 5 days [21]. The majority farmers (73.87%) discarded the spoiled WBSG and WBSY, while a small number of farmers (26.13%) reported that spoiled WBSG and WBSY was fed to dairy cattle or utilized as fertilizer. This finding is supported by Ref. [29] who reported spoiled WBSG and WBSY are used as pig feed and fertilizer.

**Table 7:** Spoilage of brewery by -products under different districts.

Questions	Response	Debre Birhan	Sululta	DebreZeit	Overall	X^2^	*P*-value
		N (%)	N (%)	N (%)	N (%)		
**Does WBSG spoil?**	Yes	53(85.48)	40(68.97)	35(87.5)	128(80)	6.99	0.03
	No	9(14.52)	18(31.03)	5(12.5)	32(20)		
**Signs of Spoilage of WBSG**	Mold	20(37.74)	15(37.5)	13(37.14)	49(38.28)	8.22	0.41
	Change in colour	5(9.43)	4(10)	4(11.43)	12(9.38)		
	Smell	14(26.42)	6(15)	8(22.86)	28(21.88)		
	Both	14(26.42)	15(37.5)	10(28.57)	39(30.47)		
**Percentage of WBSG Spoilage**	Less than 9%	25(47.17)	22(55)	21(60)	68(53.13)	29.5	0.00
	10%	12(22.64)	10(25)	4(11.43)	26(20.31)		
	11–20%	11(20.75)	5(12.5)	5(12.29)	21(14.41)		
	>20%	5(9.43)	3(7.5)	5(12.29)	13(10.16)		
**Does WBSY spoil?**	Yes	51(82.26)	51(87.93)	9(22.5)	111(69.38)	6.38	0.17
	No	11(17.74)	7(12.07)	31(77.5)	49(30.62)		
**Signs of Spoilage of WBSY**	Mold	25(49.02)	29(56.86)	2(22.22)	56(50.45)	8.58	0.38
	Change in colour	5(9.80)	7(13.73)	0(0)	12(10.81)		
	Smell	5(9.80)	3(5.88)	2(22.22)	10(9.01)		
	Both	16(31.37)	12(23.53)	5(55.56)	33(29.73)		
**Percentage of WBSY Spoilage**	Less than 9%	16(31.37)	15(29.41)	4(44.44)	35(31.53%)	10.9	0.09
	10%	12(23.53)	13(25.49)	0(0)	25(22.52)		
	11–20%	15(29.41)	12(23.53)	3(33.33)	30(27.03)		
	>20%	8(15.69)	11(21.57)	2(22.22)	21(18.92)		
**What is done with spoilt WBSG?**	Discarded	41(80.39)	36(70.59)	5(55.56)	82(73.87)	3.91	0.69
	Fed to dairy cattle	5(9.80)	5(9.80)	0(0)	10(9.01)		
	Use for fertilizer	5(9.80)	10(19.61)	4(44.44)	19(17.12)		

Wet brewer’s spent grain (WBSG); wet brewer’s spent yeast (WBSY) N = number of respondents, χ2 = Chi-square; * = significant if p < 0.05 level of significance.

### 3.8 Hygienic and safety procedures and spoilage reasons of the brewery by-products

As shown in Table 8, the majority of farmers (87.8%) said that storage duration and storage condition (temperature, moisture, humidity) are the main causes of WBSG spoilage, while fewer farmers (12.2%) said that poor hygiene in feeding troughs, transport, and storage facilities can also contribute to the spoilage of WBSG and WBSY. According to the majority of farmers (72.3%), the main hygienic and safety practices that are followed to prevent spoilage are to give fresh WBSG and WBSY without any further storage, regularly monitor the hygienic conditions of storage facilities, and use preserved WBSG. In terms of numbers, WBSG and WBSY spoilage is primarily caused by storage duration in Debre Birhan and DebreZeit town at Ada’a districts as opposed to Sululta district, while storage condition (temperature, moisture, and humidity) is indicative of brewery by product spoilage in Sululta district as compared to the other districts. Using conserved WBSG and WBSY and routinely monitoring the hygienic conditions of storage facilities indicates the main hygienic procedure of WBSG and WBSY in a larger percentage at Sululta district than Debre Birhan and DebreZeit town at Ada’a districts. This result is in line with the finding of [[29], [30], [31], [32]]; and [8] who reported using inappropriate or dirty vehicles, poor hygienic design, lack of storage and conservation facilities, brew house vessels and canning or kegging materials causes product waste and spoiling.

**Table 8:** Hygienic and safety procedures and spoilage reasons of the brewery by-products.

Questions	Response	Debre Birhan	Sululta	Debre Zeit	Overall	X^2^	*P*-value
		N (%)	N (%)	N (%)	N (%)		
**Reasons for the spoilage?**	Storage condition (temperature, moisture, humidity)	24(40.7)	23(46.9)	17(42.5)	64(43.2)	6.59	0.58
	Storage duration	29(49.2)	18(36.7)	19(47.5)	66(44.6)		
	Contamination of the feeding troughs	3(5.1)	4(8.2)	0(0)	7(4.7)		
	Contamination of the transport facilities	1(1.7)	3(6.1)	3(7.5%)	7(4.7)		
	Contamination of storage facilities	2(3.4)	1(2)	1(2.5%)	4(2.7)		
**Hygienic & safety procedures**	Use conserved grain & monitor storage facility	31(54.4)	37(63.8)	11(27.5)	79(51)	20.6	0.002
	Feed fresh by-products	8(14)	13(22.4)	12(30)	33(21.3)		
	Selectively feed/avoiding mold	6(10.5%)	2(3.4)	10(25)	18(11.6)		
	Monitoring storage & feeding facility	12(21.1)	6(10.3)	7(17.5)	25(16.1)		

N = number of respondents, χ2 = Chi-square; * = significant if p < 0.05 level of significance.

### 3.9 Constraints of brewery by-products utilization

The brewery by-product usage constraints are indicted in Table 9. The shortages of WBSG and WBSY during the fasting time associated with high purchasing costs are the major brewery by-product usage constraints. In line with this finding, shortage of WBSG and WBSY, and increasing feed costs are the constraints of dairy production in Gondar [33], Ejisu-Juaben Municipality Pig farm [29], in Sebeta, Bedele, and Debre Birhan districts [1], Hosanna town [34], West Hararghe [35] and Dilla Zuria district [ [14]]. Moreover, depending on the distance from a brewery factory and water content in WBSG can limit their availability for dairy farmers [30] and lack of knowledge, poor infrastructures, high cost of transportation and material, and the insufficient supply of the material are the major constraints to adopt agro-industrial by-products [8].

**Table 9:** Indices (weighted averages) of the major constraints of brewery by -products utilization.

Constraint	Index
**Shortage of the supply (fasting time)**	0.31
**High cost**	0.28
**Transportation problem/accessibility**	0.16
**Unscheduled selling/random selling**	0.11
**Broker problems**	0.12
**Weighting problem**	0.09

### 3.10 Major health problems in feeding brewery by-products

As shown in Table 10, some farmers reported that feeding the brewery by-products for dairy cattle causes health problems. Out of all farmers, 71 (44.38%) and 67 (41.86%) farmers reported that feeding WBSG and WBSY to dairy cattle has an effect on their health, respectively. Information of microbiota count in brewers’ spent grain is important to minimizing health risks [36]. As reported by the respondents (>15%), emaciation, diarrhea, and bloating are the major health problems caused by feeding the brewery by-products. Previous studies reported that stillbirth, abortions, delayed oestrous, bloating, diarrhea, blindness, and weak newly born calves were the main health risks related to feeding brewery spent grain [1,13,29].

**Table 10:** Major health problems in feeding brewery by-products as reported by dairy farmers.

Types of brewery by- products	Is the by-product disease source?	Health problems	Debre Birhan	Sululta	DebreZeit	Overall	X^2^	*P*-value
			N (%)	N (%)	N (%)	N (%)		
**WBSG**	Yes	–	31(50)	19(32.76)	21(72.5)	71(44.38)	5.03	0.08
	No	–	31(50)	39(67.24)	19(27.5)	89(55.62)		
		Blindness	3(9.68)	2(10.53)	0(0)	5(7.04)	4.23	0.05
		Emaciation	6(19.35)	6(31.58)	3(14.29)	15(21.13)		
		Bloating	4(12.90)	2(10.53)	4(19.05)	10(14.08)		
		Diarrhea	5(16.13)	2(10.53)	3(14.29)	10(14.08)		
		Abortion	5(16.13)	0(0)	2(9.52)	7(9.86)		
		Coughing	2(6.45)	2(10.53)	3(14.29)	7(9.86)		
		Both	6(19.35)	5(26.32)	6(28.57)	17(23.94)		
**WBSY**	Yes	–	38(62.29)	10(12.24)	19(27.5)	67(41.86)	6.65	0.05
	No	–	24(38.71)	48(82.76)	21(72.5)	89(58.14)		
		Blindness	4(10.53)	0(0)	2(10.53)	6(8.96)	5.67	0.04
		Emaciation	10(26.32)	3(30)	2(10.53)	15(22.39)		
		Bloating	10(26.32)	2(20)	4(21.05)	16(23.88)		
		Diarrhea	8(21.05)	1(10)	4(21.05)	13(19.40)		
		Both	6(15.79)	4(40)	7(36.84)	17(25.37)		

Wet brewer’s spent grain (WBSG); wet brewer’s spent yeast (WBSY) N = number of respondents, χ2 = Chi-square; * = significant if p < 0.05 level of significance.

## 4 Conclusion

Animals can be fed wet brewer's spent grain (WBSG) and wet brewer's spent yeast (WBSY) mixed with concentrate and roughage feeds. WBSG and WBSY can be preserved using a variety of processes, including as ensiling and soaking with salt, which are the common practices among dairy producers. WBSG and WBSY spoilage is caused by unhygienic conditions in feeding troughs, transport, and storage facilities. In summary, there were several factors that hindered the use of WBSG and WBSY, including availability, brokers, high cost of transportation and material, rotting, and health problems. Some of the suggested solutions are avoiding brokers, controlling cost of the WBSG and WBSY, and preserving the by-product through sun-drying and ensiling. Additionally, more study is required to determine the negative health impact of feeding WBSG and WBSY for dairy cattle.

## Declarations

### Author contribution statement

Geberemariyam Terefe, Getu Kitaw: Conceived and designed the experiments; Performed the experiments; Contributed reagents, materials, analysis, Tools or data; Wrote the paper.

Mesfin Dejene, Dereje Fekadu, Aemiro Kihalew, Bethlehem Mekonnen, and Mulugeta Walelgne: Analyzed and interpreted the data, materials, analysis tools, Wrote the paper.

### Funding statement

This research funded by Ethiopian Institute of Agricultural Research

### Data availability statement

Data will be made available on request.

### Declaration of interest’s statement

The authors declare no conflict of interest.

### Additional information

No additional information is available for this paper.
